# Barcoding of the Genus *Culicoides* (Diptera: Ceratopogonidae) in Austria—An Update of the Species Inventory Including the First Records of Three Species in Austria

**DOI:** 10.3390/pathogens9050406

**Published:** 2020-05-23

**Authors:** Carina Zittra, Günther Wöss, Lara Van der Vloet, Karin Bakran-Lebl, Bita Shahi Barogh, Peter Sehnal, Hans-Peter Fuehrer

**Affiliations:** 1 Institute of Parasitology, Department of Pathobiology, University of Veterinary Medicine, 1210 Vienna, Austria; carina.zittra@univie.ac.at (C.Z.); laruschka@gmx.at (L.V.d.V.); Karin.Bakran-Lebl@vetmeduni.ac.at (K.B.-L.); Bita.ShahiBarogh@vetmeduni.ac.at (B.S.B.); 2 Unit Limnology, Department of Functional and Evolutionary Ecology, University of Vienna, 1090 Vienna, Austria; 3 Zoological Department 2, Natural History Museum Vienna, 1010 Vienna, Austria; g.woess@gmail.com (G.W.); peter.sehnal@nhm-wien.ac.at (P.S.)

**Keywords:** biting midges, vector, mitochondrial cytochrome oxidase subunit I, *C. gornostaevae*, *C. griseidorsum*, *C. pallidicornis*

## Abstract

Ceratopogonidae are small nematoceran Diptera with a worldwide distribution, consisting of more than 5400 described species, divided into 125 genera. The genus *Culicoides* is known to comprise hematophagous vectors of medical and veterinary importance. Diseases transmitted by *Culicoides* spp. Such as African horse sickness virus, Bluetongue virus, equine encephalitis virus (Reoviridae) and Schmallenberg virus (Bunyaviridae) affect large parts of Europe and are strongly linked to the spread and abundance of its vectors. However, *Culicoides* surveillance measures are not implemented regularly nor in the whole of Austria. In this study, 142 morphologically identified individuals were chosen for molecular analyses (barcoding) of the mitochondrial cytochrome c oxidase subunit I gene (mt COI). Molecular analyses mostly supported previous morphologic identification. Mismatches between results of molecular and morphologic analysis revealed three new *Culicoides* species in Austria, *Culicoides* *gornostaevae* Mirzaeva, 1984, which is a member of the Obsoletus group, *C. griseidorsum* Kieffer, 1918 and *C. pallidicornis* Kieffer, 1919 as well as possible cryptic species. We present here the first Austrian barcodes of the mt COI region of 26 *Culicoides* species and conclude that barcoding is a reliable tool with which to support morphologic analysis, especially with regard to the difficult to identify females of the medically and economically important genus *Culicoides*.

## 1. Introduction

Ceratopogonidae (Insecta: Diptera) are 0.5- to 3.0-mm long midges [1] with more than 5400 described species out of 125 genera and 38 groups [2,3]. In Europe, 567 ceratopogonid species are known [1]. Females of four genera, *Austroconops*, *Lasiohelea*, *Leptoconops* and *Culicoides*, are obligate hematophagous biting midges and vectors of pathogens of medical and veterinary importance or are at least known as a biting nuisance [4]. Especially members of the genus *Culicoides* are important vectors of economically important viruses such as African horse sickness (AHS) virus, Bluetongue (BT) virus, equine encephalitis virus (EEV) (Reoviridae) and Schmallenberg (SB) virus (Bunyaviridae) [5,6]. In particular, BT and SB viruses not only affect ruminants, but also new world camelids, whose abundance is steadily increasing in Austria, are susceptible to viral pathogens and should be considered as carriers and reservoirs [7,8]. Recently, SB virus antibodies were found in horses in Iran [9], revealing a possible unrecognized reservoir for this virus. Furthermore, *Culicoides* spp. cause common allergic dermatitis in Icelandic horses and insect bite hypersensitivity (IBH), also known as sweet itch [10]. The hibernation of the BT virus in *Culicoides* is still a frequently discussed topic. Male as well as gravid, parous and nulliparous females were found beyond the usual activity period between spring and autumn, but also during the winter season when optimal conditions prevail [11]. Moreover, successful hibernation of the BT virus with a subsequent spread in the next spring has already been observed [12].

Diseases transmitted by *Culicoides* spp. affect large parts of Europe and are strongly linked with the spread and abundance of their vectors [13]. Nevertheless, *Culicoides* surveillance in Austria (apart from a single Bluetongue surveillance program summarized in Anderle et al. [11]) is mainly implemented on a small scale, where opportunities for cost-effective continuous sampling exists [14].

The genus *Culicoides* Latreille, 1809, is distributed worldwide and includes 1365 species [2], of which 129 are confined to Europe [15], and of which species of the Obsoletus group seem to be most abundant [13]. Approximately 30 species are capable of BT virus transmission, at least under laboratory conditions [16]. Proven vectors of BT virus are *C. imicola*, *C. brevitarsis*, *C. bolitinos* (subgenus *Avaritia* Fox, 1955), *C. pulicaris* (subgenus *Culicoides*), *C. sonorensis* (subgenus *Monoculicoides* Khalaf, 1954) and *C. insignis* (subgenus *Hoffmania* Fox 1948). In the Mediterranean region only *C. obsoletus*, *C. scoticus*, *C. pulicaris* and *C. imicola* are present, with the latter accounting for approximately 90% of BT virus transmission in this region [16] In the temperate climate, *C. obsoletus* and *C. scoticus* are the most widely distributed livestock-associated *Culicoides* species [17]. *Culicoides obsoletus*, *C. scoticus* and *C. pulicaris* are also widely distributed in Austria [18,19]. At present, the Austrian species inventory consists of 32 *Culicoides* species [18], of which 19 were recorded for the first time in Austria between 2007 and 2010 within the framework of large-scale Bluetongue and *Culicoides* surveillance [11].

Initial monitoring approaches for *Culicoides* necessitate highly skilled entomologists, because species identification is impeded by a high number of cryptic species and females. Barcoding is an adequate molecular tool to supplement morphologic identification of these cryptic species or species groups and seems to be essential for further monitoring approaches. Furthermore, molecular analysis can give a first hint at revealing previously unrecognized cryptic species [20].

## 2. Results

The *Culicoides* monitoring during the Bluetongue surveillance yielded 30 species [11]. In our re-assessment of this sampling, a total of 77 sequences of the mitochondrial COI barcode region were obtained from 108 female and 34 male specimens of the genus *Culicoides* that initially were identified as belonging to 32 species, species complexes or hardly distinguishable species pairs (Table 1).

Morphologic and molecular identification was congruent in 59 specimens, i.e., suggesting the same taxonomic entities for 17 of 19 taxa (Table 2), recorded in Austria during the Bluetongue surveillance [11].

Furthermore, molecular identification revealed the presence of three species of *Culicoides* new to Austria, namely *C*. (*Avaritia*) *gornostaevae*, *C*. (*Silvaticulicoides*) *griseidorsum* and *C*. (*Wirthomyia*) *pallidicornis* (Table 1 and Table 2). Molecular identification was corroborated by morphologic analysis using the key of Mathieu et al. [22]. A total of five specimens collected in May 2009, mistakenly identified by morphology as *C. clastieri* (four females) and as *C. duddingstoni* (one female), were identified by means of molecular tools as *C. griseidorsum*. This species is known from four countries bordering Austria (Germany, Italy, Slovakia and Switzerland). A retrospective reexamination of the morphologic characters using the redescription of this taxon by Szadziewski et al. [15] also identified those specimens as *C. griseidorsum. Culicoides pallidicornis,* generally present in bordering countries of Austria (Italy, Switzerland, Germany, Slovakia, Czech Republic and Hungary), was represented by a single female, morphologically misidentified as *C. subfasciipennis,* which had been collected in Carinthia in June 2009. Subsequent morphologic identification using the key of Mathieu et al. [22] confirmed the result of the molecular analysis. The presence of *C. gornostaevae* was confirmed only by molecular tools. This member of the Obsoletus group, a possible vector for Schmallenberg and Bluetongue virus, is so far only known from Norway, Poland, Siberia and Sweden [23].

Molecular analyzes recovered some putatively cryptic species. Four of five specimens of *C. grisescens* (D24, D178, D179 and D184) were identical to a specific type, *C. grisescens* G2, in which the specimens are morphologically similar, but differ substantially in their barcode sequences [23]. The same was observed within the Pulicaris and the Obsoletus group, where several types of mt COI sequences were observed (Table 1). Furthermore, we provide here the first barcode of *C. minutissimus* (= syn. *C. pumilus*) in the BOLD sequence database, obtained from a male specimen.

Two specimens—originally identified morphologically as *C. lupicaris—*were identical as in their barcode region to the as yet undescribed lineage *Culicoides* sp. CW2011, which was initially reported in 2011 in Switzerland [21]).

## 3. Discussion

Information on seasonal and spatial distribution patterns as well as autecology of different *Culicoides* species is, with exception of some single studies [11], limited in Austria. This is mostly due to identification of *Culicoides* species to complex or group-level, but not to species-level [14], as identification of *Culicoides* spp. below group-level by morphology can be difficult or impossible if reliable morphologic characters are absent–especially in those species with weakly developed wing pattern. In addition, morphology, ecology and microhabitats of most of the immature stages of different *Culicoides* species are unknown [1].

This is the first study in Austria to investigate cryptic *Culicoides* at the species-level, which we achieved using a DNA barcoding approach, in addition to exhaustive morphologic studies. DNA barcodes are already known to be able to uncover unrecognized cryptic species, especially in the suborder Nematocera (Insecta: Diptera), as well as in Chironomidae [20], Cecidomyiidae [24] and Simuliidae [25]. To derive a valid description of these new putative species and to assign a formal scientific name based on mt COI sequence divergence, is still challenging, and complicates the interpretation of results. With respect to *C. grisescens*, only *C. grisescens* G2, a lineage initially reported in 2012 [21], was examined in our study, not the nominal species. Furthermore, two types of *C. obsoletus* and three types of *C. pulicaris* were found (Table 1). The Obsoletus group consists of the morphologically similar *C. obsoletus*, C. *scoticus*, *C. dewulfi* and *C. chiopterus*. Controversially, the term *Obsoletus* complex is mainly used for *C. obsoletus*, *C. scoticus* and *C. montanus* (a species not belonging to the Obsoletus group), in which the females cannot be distinguished by morphology. However, males of *C. obsoletus*, *C. dewulfi*, *C. chiopterus* and *C. scoticus* can be differentiated reliably by morphology and both sexes by the mt COI barcode region (Table 1). In addition to the known *Obsoletus* complex diversity, several cryptic species related to *C. obsoletus* were found in Central Europe [26]; their potential presence in Austria remains to be investigated as these are not formally described or related to particular mt COI sequences. The absence of *C. montanus* from Austria can, however, be safely assumed, as this taxon is known to be rare and restricted to the southern Mediterranean region [26]. Moreover, the first finding of *C. gornostaevae*, a boreal species belonging to the Obsoletus group, was facilitated by the analysis of the barcode region. *Culicoides gornostaevae* was previously known only from Norway, Poland, Siberia and Sweden [22]. Males of this taxon can be separated from the other members of the Obsoletus group by the genitalia armature [22]. The overall lack of reliable reference data for the Obsoletus group allows us to confirm the presence of two taxa, that may either be a member of the nominal group or represent yet unknown species.

The Pulicaris group is considered to consist of 14 distinct taxa: *C. pulicaris*, *C. lupicaris*, *C. impunctatus*, *C. punctatus*, *C. grisescens*, *C. newsteadi*, *C. flavipulicaris*, *C. fagineus*, *C. subfagineus*, *C. bysta* n. sp., *C. paradoxalis* sp. nov., *C. boyi* sp. nov., *C. selandicus* sp. nov. and *C. kalix* sp. nov. [27]. In a Turkish study, three different haplotypes of *C. lupicaris* were recovered [27,28,29,30] and results obtained here indicate a similar situation in *C. pulicaris*.

Interestingly, specimens originally identified as *C. lupicaris* (Table 1) were identical to a yet unnamed lineage first reported in 2011 from Switzerland using barcoding [21]. This is particularly intriguing as these specimens displayed the morphologic characters typical of *C. lupicaris*. These results should clearly be able to spark a taxonomic reevaluation of the Pulicaris group.

In our case morphologic identification was principally congruent with molecular analysis. Mismatched results underline the difficulties of identifying female *Culicoides*, especially in species groups or complexes, and that identification of male *Culicoides* species is less susceptible to errors than females. In-depth research needs to be done within *Culicoides* species groups to correctly differentiate the group members, at least with molecular tools. Investigations on possible differences in ecology, behavior and vector competence of those newly found cryptic species could then follow. This will also be of relevance to epidemiological research as BT viruses are still to be considered in Austria. The first BT virus case was reported in the district of Schaerding, Upper Austria in November 2008, where in total 28 BT virus serotype 8 positive animals were found; extensive vaccination measures followed [31]. The Austrian BT monitoring and vaccination programs after this outbreak led to total costs of €23.6 million [32] up until 2016. In 2015, BT serotype 4 was found for the first time in the federal states Burgenland and Styria and in 2016 a BT virus serotype 4 outbreak was reported from Carinthia [32].

Correct species identification of important vectors is crucial to assess pathogen transmission potential. Cryptic species may have different ecological niches and may differ in their epidemiological relevance, thus resolving taxonomic conundrums is necessary. In addition, molecular identification through DNA barcoding may support exploration of life cycles and life history traits in the future to better characterize breeding habitats of *Culicoides* in Austria. Misidentification of these vectors can lead to substantial epidemiological implications, such as neglect of vaccination measures and loss of livestock. We conclude that morphological identification supported by molecular analysis such as DNA barcoding bases on the mt COI gene is an adequate tool for species identification in *Culicoides*. We strongly suggest using both identification approaches to deliver high quality data –necessary for both ecological research and vector-transmitted disease risk assessment. However, use of molecular tools alone is not recommended due to shortcomings of available reference libraries, which can hamper identification. Additionally, employing sets of different primers is strongly recommended within the order Diptera. General primers often fail to amplify all taxa within a family, e.g., mosquitoes, in which sets of more specific primers should be used [33]. Moreover, barcoding fails at separating the species within certain complexes (e.g., *Anopheles maculipennis* complex). In the present study, we used three different primer pairs to increase the success rate of amplification and subsequent identification. Species identification of larvae is virtually impossible as larvae lack reliable morphologic characteristics [34]. At present, larvae of most Ceratopogoninae and Palpomyiinae species are not described at species-level [1]. Only 13% of the larvae and 17% of the pupae of the genus *Culicoides* are known, but often poorly described [3]. Currently, successful hatching of larvae collected to identify the adults is a common identification strategy [35]. Molecular tools may open the way to a more comprehensive investigation of this important taxon. Accelerated identification by means of molecular barcodes will therefore support future studies on larval micro-habitats—knowledge which will be crucial for the development of preventive measures of *Culicoides*-borne diseases or the prevention of creating larval micro-habitats around farms where emerging disease outbreaks may happen [36].

This study provides the first barcodes for *Culicoides* biting midges and the first record of *Culicoides gornostaevae*, *C. griseidorsum* and *C. pallidicornis* in Austria. We demonstrate that morphologic identification is primarily congruent with molecular analyzes. However, intense taxonomic, epidemiological and ecological research efforts, ideally supported by molecular tools, are still necessary to differentiate species based on their ecology, behavior and vector competence.

## 4. Materials and Methods

*Culicoides* were sampled within a Bluetongue virus monitoring program carried out from 2007 to 2010 and realized in all Austrian provinces [11] using Onderstepoort-type black light traps [11,37] which were set weekly at a total of 54 sampling sites. Individuals were separated by sex and identified to species- or at least to group-level by morphology using the key of Delécolle [38] and were stored in 75% ethanol (EtoH). One to eight well preserved specimens from each species were sampled from different provinces and used for barcoding. After photographic documentation, DNA was extracted from one to three legs of each individual. Three 1.4 mm ceramic beads (Precellys Ceramic Kit 2.8 mm/1.4 mm, Peqlab, Erlangen, Germany) were added to each tissue sample. After homogenization with TissueLyser II (Qiagen, Hilden, Germany), DNA was extracted using the “DNeasy^®^ Blood and Tissue” DNA isolation kit according to the manufacturer’s protocol (Qiagen, Hilden, Germany). Conventional polymerase chain reaction (PCR), targeting an approximately 667 bp fragment of the mitochondrial cytochrome c oxidase subunit I gene (COI) within the BOLD-barcode region using primers H15CuliCOIFw and H15CuliCOIRv, LCO1490 and HCO2198 as well as LepF1 and LepR1 was performed as reported previously [30,39,40,41]. After molecular specification, *Culicoides* specimens with mismatching results in morphologic and molecular features were rechecked using an up to date online identification key for female *Culicoides* from the West Palearctic region (IIKC) by Mathieu et al. [21], which was not available at the sampling time.

Finally, morphologically and molecularly identified voucher specimens were deposited in the Diptera collection of the Natural History Museum Vienna.

## Figures and Tables

**Table 1:** *Culicoides* taxa identified by morphology and mt mitochondrial cytochrome c oxidase subunit I gene (mt COI) including sampling date, location and storage conditions.

Taxon, ID, Bold ID	Sex	Collection Date	Province	Site	Max.% Identity to GenBank Entries	Max.% Identity to Bold Entries
*Culicoides chiopterus* (D155) GNIA001-20	M	21.09.2009	S	Zell/See	100% (JQ898006.1)	100% (GBDP11539-12.COI-5P)
*C. chiopterus* (D157) GNIA002-20	F	14.06.2010	ST	Knittelfeld	100% (JQ898010.1)	100% (GBMIN29061-13.COI-5P)
*C. comosioculatus* (D84) GNIA003-20	F	07.06.2010	UA	Gmunden	100% (HQ824466)	100% (GBDP11574-12.COI-5P)
*C. deltus* (D139) GNIA004-20	F	28.06.2009	LA	Hollabrunn	99.52% (JF766303)	100% (GBMIN29039-13.COI-5P)
*C. deltus* (D197) GNIA005-20	M	27.07.2009	S	Zell/See	100% (HQ824455)	100% (GBMIN29039-13.COI-5P)
*C. dewulfi* (D147) GNIA006-20	M	21.09.2009	S	Zell/See	100% (KF802203)	100% (GBDP11548-12)
*C. dewulfi* (D148) GNIA007-20	M	21.09.2009	S	Zell/See	100% (KF802203)	100% (GBDP11548-12)
*C. dewulfi* (D180) GNIA008-20	M	27.07.2009	S	Zell/See	100% (KT186808)	100% (GBMIN29059-13)
*C. dewulfi* (D188) GNIA009-20	F	07.06.2009	UA	Braunau	100% (JQ897994)	100% (GBMIN29059-13)
*C. fascipennis* (D101) GNIA010-20	F	18.05.2009	V	Favoriten	100% (JQ620075)	100% (GBDP11581-12)
*C. fascipennis* (D103) GNIA011-20	F	03.07.2009	V	Favoriten	100% (KJ767936	100% (GBDP11582-12)
*C. fascipennis* (D202) GNIA012-20	F	27.07.2009	S	Zell am See	100% (KJ767936)	100% (GBDP11582-12)
*C. festivipennis* (D113) GNIA013-20	F	13.07.2009	C	Spittal/Drau	99.77% (HM241866)	99.54% (GBDP11586-12)
*C. festivipennis* (D114) GNIA014-20	F	29.06.2009	UA	Urfahr-Umgebung	99.77% (HM241866)	99.54% (GBDP11586-12)
*C. festivipennis* (D207) GNIA015-20	F	01.06.2009	S	Spittal/Drau	99.1% (HM241866)	99.54% (GBDP11586-12)
*C. festivipennis* (D208) GNIA016-20	F	01.06.2009	C	Spittal/Drau	100% (JQ620084)	99.55% (GBDP11586-12)
*C. festivipennis* (D209) GNIA017-20	F	01.06.2009	C	Spittal/Drau	100% (HM241866)	99.77% (GBDP11586-12)
*C. furcillatus* (D100) GNIA018-20	F	03.07.2009	C	Spittal/Drau	99.79% (KJ624083)	99.77% (GBDP11592-12)
*C. furcillatus* (D128) GNIA019-20	F	29.06.2009	UA	Urfahr-Umgebung	nd	100% (GBDP11592-12)
*C. furcillatus* (D102) GNIA020-20	F	03.07.2009	C	Spittal/Drau	100% (KJ624083)	100% (GBDP11591-12)
*C. furcillatus* (D133) GNIA021-20	F	17.08.2009	UA	Urfahr-Umgebung	100% (KJ624083)	100% (GBDP11592-12)
*C. furcillatus* (D201) GNIA022-20	F	27.07.2009	S	Zell am See	100% (KJ624083)	100% (GBDP11592-12)
*C. gornostaevae* (D28)* GNIA023-20	F	07.06.2010	LA	Zwettl	100% (JQ620138)	100% (GMGRD2770-13)
*C. griseidorsum* (D104)* GNIA024-20	F	18.05.2009	V	Favoriten	100% (MN274523)	nd
*C. griseidorsum* (D106)* GNIA025-20	F	18.05.2009	V	Favoriten	100% (MN274523)	nd
*C. griseidorsum* (D107)* GNIA026-20	F	18.05.2009	B	Neusiedl/See	99.8% (MN274523)	nd
*C. griseidorsum* (D111)* GNIA028-20	F	11.05.2009	LA	Gänserndorf	99.83% (MN274523)	nd
*C. griseidorsum* (D138)* GNIA029-20	F	18.05.2009	V	Favoriten	100% (MN274523)	nd
*C. grisescens* (D145) 1 GNIA044-20	M	21.09.2009	S	Zell/See	98.52% (KJ767938)	98.9% (early release)
*C. grisescens* (D146) GNIA045-20	M	21.09.2009	S	Zell/See	99.85% (KJ767938)	100% (FICER050-12)
*C. grisescens* (D178) ^1^ GNIA046-20	F	27.09.2009	S	Zell am See	99.32% (HQ824453)	99.32% (GBMIN29040-13)
*C. grisescens* (D179) ^1^ GNIA047-20	F	27.09.2009	S	Zell/See	99.77% (HQ824452)	99.77% (GBDP32600-19)
*C. grisescens* (D184) ^1^ GNIA048-20	F	21.09.2009	ST	Mürzzuschlag	99.77% (HQ824452)	99.77% (GBDP32600-19)
*C. grisescens* (D24) ^1^ GNIA049-20	F	21.09.2009	S	Zell/See	99.77% (HQ824452)	99.77% (GBDP32600-19)
*C. kibunensis* (D183) GNIA050-20	F	02.06.2008	V	Favoriten	99.58% (KJ624094)	99.85% (GMGRD1621-13)
*C. kibunensis* (D34) GNIA051-20	F	07.06.2009	LA	Zwettl	100% (JQ683272)	99.08 (private)
*C. lupicaris* (D74^)1^ GNIA052-20	F	07.06.2010	S	Tamsweg	100% (HQ824440)	100% (GBDP11558-12)
*C. lupicaris* (D75) ^1^ GNIA05320	F	07.06.2010	S	Tamsweg	98.64% (HQ824442)	98.85% (GBDP11562-12)
*C. lupicaris* (D57) GNIA063-20	M	03.05.2010	UA	Kirchdorf/ Krems	100% (KF591605)	99.84 (GBDP33078-19)
*C. lupicaris* (D58) GNIA064-20	M	03.05.2010	UA	Kirchdorf/ Krems	100% (KF591605)	99.84 (GBDP33078-19)
*C. minutissimus* (D191) GNIA055-20	M	24.08.2009	LA	Tulln	no entry	no entry
*C. nubeculosus* (D124) GNIA065-20	F	10.08.2009	LA	Gänserndorf	nd	nd
*C. nubeculosus* (D125) GNIA066-20	F	10.08.2009	LA	Gänserndorf	nd	nd
*C. obsoletus* (D45) GNIA074-20	M	25.05.2009	C	Villach	100% (HM022818)	100% (GMGRE3865-13)
*C. obsoletus* (D46) GNIA075-20	M	25.05.2009	C	Villach	100% (HQ824383)	100% (GBMIN29075-13)
*C. obsoletus* (D48) GNIA076-20	M	28.06.2010	ST	Liezen	100% (KT186816)	100% (GMGRE3865-13)
*C. pallidicornis* (D35)* GNIA071-20	F	07.06.2010	LA	Zwettl	99.36% (KJ624111)	100% (early release)
*C. pallidicornis* (D193)* GNIA072-20	M	01.06.2009	C	Spittal/Drau	100% (JQ620154)	100% (GBDP11602-12)
*C. pallidicornis* (D198)* GNIA073-20	F	01.06.2009	C	Spittal/Drau	100% (JQ620154)	100% (GBDP11602-12)
*C. pictipennis* (D149) GNIA069-20	F	15.06.2009	T	Reutte	99.78% (JQ620162)	100% (private)
*C. pictipennis* (D151) GNIA070-20	F	11.05.2009	B	Güssing	99.78% (JQ620162)	100% (private)
*C. poperinghensis* (D181) GNIA067-20	F	14.05.2010	V	Favoriten	100% (JQ620166)	99.6% (private)
*C. poperinghensis* (D182) GNIA068-20	F	02.06.2008	V	Favoriten	100% (JQ620166)	99.6% (private)
*C. pulicaris* (D49) GNIA060-20	F	10.05.2010	LA	Zwettl	100% (JQ620183)	99.76% (GBDP11551-12)
*C. pulicaris* (D51) GNIA061-20	F	21.09.2010	UA	Perg	100% (AM236711)	100% (GBMIN17621-13)
*C. pulicaris* (D54) GNIA062-20	M	14.06.2010	ST	Bruck/Mur	100% (JF766336)	99.76% (GBDP11551-12)
*C. punctatus* (D129) GNIA055-20	F	18.05.2009	V	Favoriten	100% (MN274527)	98.77% (private)
*C. punctatus* (D130) GNIA056-20	F	18.05.2009	V	Favoriten	99.68% (MN274527)	99.69% (private)
*C. punctatus* (D159) GNIA057-20	F	08.06.2009	LA	Gänserndorf	99.36% (MN274527)	99.39% (private)
*C. punctatus* (D160) GNIA058-20	F	15.06.2009	T	Reutte	100% (KY707780)	100% (GBDP11610-12)
*C. punctatus* (D162) GNIA059-20	M	17.08.2009	UA	Urfahr-Umgebung	99.78% (KY707779)	98.62% (private)
*C. reconditus* (D186) GNIA039-20	F	10.08.2009	UA	Linz-Land	98.48% (KJ767956)	98.4% (FICER086-12)
*C. reconditus* (D192) GNIA040-20	F	27.07.2009	S	Tamsweg	98.48% (KJ767956)	98.4% (FICER086-12)
*C. reconditus* (D194) GNIA041-20	F	17.08.2009	S	Tamsweg	100% (JQ620193)	98.78% (early release)
*C. reconditus* (D195) GNIA042-20	F	17.08.2009	S	Tamsweg	99.77% (HQ8245089	99.77% (GBDP11615-12)
*C. reconditus* (D199) GNIA043-20	F	24.08.2009	C	Spittal/Drau	99.77% (HQ824508)	99.76% (GBDP11615-12)
*C. riethi* (D108) GNIA027-20	M	14.09.2009	B	Neusiedl/See	99.82% (MN274531)	nd
*C. riethi* (D121) GNIA030-20	M	13.07.2009	LA	Mistelbach	100% (JQ620196)	nd
*C. riethi* (D126) GNIA031-20	F	10.08.2009	LA	Mistelbach	100% (JQ620196)	nd
*C. riethi* (D127) GNIA032-20	F	10.08.2009	LA	Mistelbach	100% (JQ620195)	nd
*C. riouxi* (D187) GNIA033-20	F	10.08.2009	LA	Melk	100% (KJ624124)	98.7% (private)
*C. salinarius* (D154) GNIA034-20	F	29.06.2009	LA	Hollabrunn	100% (JQ620198)	98.5% Culicoides sp. (CNGSD4978-15)
*C. salinarius* (D190) GNIA035-20	F	06.07.2009	St	Feldbach	99.57% (KJ624125)	99.64% (early release)
*C. scoticus* (D44) GNIA036-20	M	03.08.2010	ST	Liezen	100% (KT186879)	100% (GBMIN29072-13)
*C. scoticus* (D144) GNIA037-20	M	29.06.2009	UA	Urfahr-Umgebung	100% (KT186879)	100% (GBMIN29072-13)
*C. subfasciipennis* (D177) GNIA038-20	F	23.06.2008	LA	Scheibbs	99.14% (JQ620238)	98.92% (private)

Abbreviations: F = female; M = male; S= Salzburg; St = Styria; UA = Upper Austria; V = Vienna; C = Carinthia; B = Burgenland; LA = Lower Austria; nd = no data for specification; * first record for Austria; ^1^ cryptic species. Wenk et al. [21].

**Table 2:** Updated list of the Austrian *Culicoides* species inventory representing 36 species.

Taxon (Author, Year)
*C. alazanicus* (syn. of *C. musilator*) (Dzhafarov 1961)
*C. albicans* (Winnertz 1852) +
*C. chiopterus* (Meigen 1830)
*C. circumscriptus* (Kieffer 1918) +
*C. clastrieri* (Callot, Kremer and Deduit 1962) +
*C. comosioculatus* (syn. of *C. chetophthalmus*) (Tokunaga, 1956) +
*C. deltus* (Edwards 1939) +
*C. dewulfi* (Goetghebuer 1933) +
*C. duddingstoni* (Kettle and Lawson, 1955) +
*C. fascipennis* (Staeger 1839)
*C. festivipennis* (Kieffer 1914)
*C. furcillatus* (Callot, Kremer and Paradis 1962) +
*C. griseidorsum* (Kieffer 1918) *
*C. grisescens* (Goetghebuer 1935) +
*C. gornostaevae* (Mirzaeva, 1984) *
*C. kibunensis* (Tokunaga, 1937)
*C. lupicaris* (Downes and Kettle, 1952)
*C. minutissimus* (Zetterstedt 1855)
*C. newsteadi* (Austen 1921) +
*C. nubeculosus* (Tokunaga 1941)
*C. obsoletus* (Meigen 1818)
*C. pallidicornis* (Kieffer, 1919) *
*C. pictipennis* (Staeger 1839)
*C. poperinghensis* (Goetghebuer 1953) +
*C. pulicaris* (Linnaeus 1758)
*C. punctatus* (Meigen 1804) +
*C. reconditus* (Campbell and Pelham-Clinton 1960) +
*C. riethi* (Kieffer, 1914) +
*C. riouxi* (Huttel and Huttel 1951) +
*C. saevus* (Kieffer, 1922)
*C. salinarius* (Kieffer, 1914) +
*C. scoticus* (Downes and Kettle, 1952) +
*C. segnis* (Campbell and Pelham-Clinton 1960) +
*C. stigma* (Meigen 1818)
*C. subfasciipennis* (Kieffer 1919)
*C. vexans* (Staeger 1839)

* First record for Austria in the framework of this study; + first record for Austria in Anderle et al. [11].
